#  Design, Synthesis and Anti-Tubercular Activity of Novel 1, 4-Dihydropyrine-3, 5-Dicarboxamide Containing 4(5)-Chloro-2-Ethyl- 5(4)-Imidazolyl Moiety

**Published:** 2016

**Authors:** Maryam Iman, Asghar Davood, Mahboubeh Lotfinia, Golnoush Dehqani, Soroush Sardari, Parisa Azerang, Mohsen Amini

**Affiliations:** a *Chemical Injuries Research Center, Baqiyatallah University of Medical Sciences, Tehran, Iran.*; b *Department of Medicinal Chemistry, Faculty of Pharmacy, Pharmaceutical Sciences Branch, Islamic Azad University, Tehran, Iran (IAUPS). *; c *Department of Bioinformatics and Drug design, Institute Pasteur, Tehran, Iran. *; d *Department of Medicinal Chemistry, Faculty of Pharmacy, Tehran University of Medical Sciences, Tehran, Iran.*

**Keywords:** Dihydropyridine, Mycobaterium, Tuberculosis, Imidazole

## Abstract

Current researches have showed that N3, N5-diaryl-2, 6-dimethyl -1, 4-dihydropyrine-3, 5- dicarboxamide analogues demonstrate notable anti-tubercular activity. In this study, Hantzsch condensation was used to design and synthesize new analogues of dihydropyridine (DHP). Different diary carboxamides were inserted at positions 3 and 5 of the DHP ring. 4(5)-chloro-2-ethyl-5(4)-imidazolyl moiety was considered at position 4 of the DHP ring. The structures of prepared ligands were characterized using TLC followed by FT-IR, elemental analysis, Mass and proton NMR. Results of anti-tubercular activity have indicated all the prepared ligands 3a-f inhibit the mycobacterium tuberculosis growth and the most potent compounds were 3c (3,4-Cl) and 3b (4-Cl). The *in-vitro* obtained data are agreement with our computational predictions in terms of partial atomic charge of carbonyl moieties at the positions 3 and 5 of dihydropyridine ring and the logP of the molecules.

## Introduction

After AIDS, tuberculosis (TB) is one of the most common reasons of death from infectious disease and around 1/3 of the world’s population are infected with mycobacterium tuberculosis (1). About 1% of the population is infected with TB each year (2). Since the high rate of MDR-TB and the co-infection with HIV are the main problem in the treatment of TB, the finding the new and efficient anti-TB ligand is required. Since the mid-1990s the publication and research in TB has increased that reported novel molecules as potential leads for TB drug discovery (3-8).

1, 4-Dihydropyridine (DHP) acts as calcium channel modulators (9-14), and also is a fruitful lead compound that shows several biological activities. It has been shown that the substitution of a carboxylate ester with an aryl carboxamide group in 1,4-dihydropyridines provided significant antitubercular properties (15-19). 

**Figure 1:**
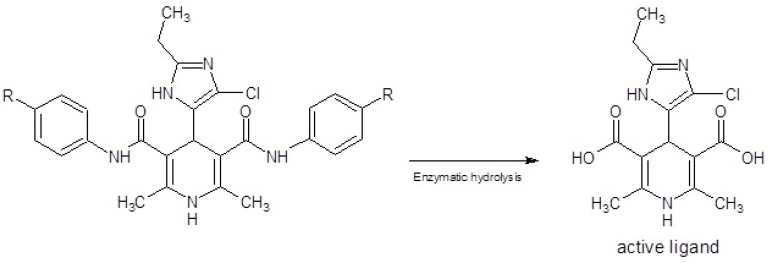
Enzymatic bio-activation of dihydropyridines 3a-f

Using the aryl amide in the positions 3 and 5 of DHPs result in to increase the anti-tubercular activity and reduce the calcium channel modulatory activities (20-23). Lipophilicity of DHP and partial atomic charge (PAC) of carboxamide moiety affect the penetration into mycobacterium cells and subsequently enzymatic activation respectively (27-29). This type of DHPs acts as prodrug, and within the mycobacterium cell wall, carboximide groups hydrolysis to active carboxylic acid groups (24-26) (Figure 1.), so anti-tubercular activity of this series of ligands can be affected mainly by the aryl moieties in the position 4 of DHPs. It was revealed that using N1-substituted imidazole moieties with an electron donating group (alkylthio), in the 4 position of DHPs, result in to decrease the anti-tubercular activity (19). Due to this negative affect of π-excessive and N-substituted imidazole moiety and based on our previous studies (11-16) that have confirmed imidazole is tolerated in active site of DHPs (5-9), it is suggested that using the N-unsubtituted imidazole which contain an electron withdrawing group (Cl), can increase the anti-tubercular activity. So here in as a part of our ongoing research we designed and synthetized novel DHPs as an anti-tubercular agents using 4(5)-chloro-2-ethyl-5(4)-imidazolyl and aryl amide moieties in the C-4, C-3 and C-5 of DHP ring in which the both of nitrogen atoms of imidazole moiety are unsubstituted and it was slightly π-deficient.

## Material and Methods

*Chemistry*

All of the prepared ligands were characterized using thin layer chromatography followed by FT-IR, Mass, elemental analysis and H-NMR. Melting points were firmed using a Thomas- Hoover capillary apparatus and were uncorrected. 1HNMR spectra were recorded on a Bruker FT-250 spectrometer TMS was used as an internal standard. Infrared spectra were acquired on a Nicolet 550-FT spectrometer. Mass spectra were measured with a Finnigan TSQ-70 spectrometer (Finnigan Mat, Bremen, Germany). Elemental analysis was done using a Perkin-Elmer model 240-C apparatus. The results of elemental analysis (C, H, and N) were within ± 0.4% of the calculated amounts. Reagents and solvents were prepared from MERCK (Darmstadt, Germany). DHPs 3a-f (Table 1.) were produced using Hantzsch condensation (30, 31) in which 4(5)-chloro-2-ethylimidazole-5(4)-carboxaldehyde 2 was condensed with N-arylacetoacetamide 1a-f and ammonium acetate in methanol (Figure 1). The compound 2 could be prepared in three-step from propionaldehyde, dihydroxyacetone and ammonia 5 3-oxo-N-aryl butanamide 1a-f was produced according to modified Clemens method33 by condensation of 2, 2, 6-trimethyl-1, 3-dioxin-4-one with the appropriate arylamine.

General procedure for the preparation of 3-oxo-N-aryl butanamide (1a-g) 

A solution of an appropriate arylamine and 2, 2, 6-trimethyl-1, 3-dioxin-4-one in xylene was placed in an Erlenmeyer flask. The flask was immersed in an oil bath that had been preheated to 150 °C, and the solution was vigorously stirred. The evolution of acetone became apparent within several minutes and heating was continued for 3-4 h. The xylene was then removed, and the product was filtered and recrystallized from appropriate organic solvents.

*N-(4-fluorophenyl)-3-oxobutanamide (1a)*

Using the general procedure and 4-fluoroaniline provided the title compound after 3 h of reflux: White crystals (from chloroform and petroleum ether; 1:1), Yield 88%, mp 98-101 °C. IR (KBr): ν 3315(NH), 1716, 1671 cm^−1^ (CO). ^1^H-NMR (CDCl_3_): δ (ppm) 2.29 (s, 3H, CH_3_), 3.70 (s, 2H, CH_2_), 7.15-7.25 (m, 2H, H-3, 5-phenyl), 7.56-7.78 (m, 2H, H-2, 6-phenyl), 9.40 (brs, 1H, NH).

*N-(4-chlorophenyl)-3-oxobutanamide (1b)*

Using the general procedure and 4-chloroaniline provided the title compound after 3 h of reflux: White crystals (from chloroform and petroleum ether; 1:1), Yield 79%, mp 127-129 °C. IR (KBr): ν 3300(NH), 1720, 1670 cm^−1^ (CO). ^1^H-NMR (CDCl_3_): δ (ppm) 2.30 (s, 3H, CH_3_), 3.58 (s, 2H, CH_2_), 7.23 (d, J=8 Hz, 2H, H-3,5-phenyl), 7.50 (d, J=8 Hz, 2H, H-2,6-phenyl), 9.37 (s, 1H, NH). 

*N-(3, 4-dichlorophenyl)-3-oxobutanamide (1c)*

Using the general procedure and 3, 4-dichloroaniline provided the title compound after 4 h of reflux: White crystals (from chloroform and petroleum ether; 1:1), Yield 65%, mp 79-81 °C. IR (KBr): ν 3378(NH), 1710, 1665 cm^−1^ (CO). ^1^H-NMR (CDCl_3_): δ (ppm) 2.39 (s, 3H, CH_3_), 3.65 (s, 2H, CH_2_), 7.30-7.47 (m, 2H, H-5, 6-phenyl), 8.05 (s, 1H, H-2-phenyl), 9.45 (brs, 1H, NH).

*N-(3-bromophenyl)-3-oxobutanamide (1d)*

Using the general procedure and 3-bromoaniline provided the title compound after 3 h of reflux: Light yellow crystals (from chloroform and petroleum ether; 1:1), Yield 80%, mp 98-101 °C. IR (KBr): ν 3300(NH), 1712, 1673 cm^−1^ (CO). ^1^H-NMR (CDCl_3_): δ (ppm) 2.29 (s, 3H, CH_3_), 3.54 (s, 2H, CH_2_), 7.18-7.45 (m, 3H, H-4, 5, 6-phenyl), 7.78 (s, 1H, H-2-phenyl), 9.25 (brs, 1H, NH). 

*N-(2-nitrophenyl)-3-oxobutanamide (1e)*

Using the general procedure and 2-nitroaniline provided the title compound after 4 h of reflux: Yellow crystals (from petroleum ether), Yield 70%, mp 142-143 °C. IR (KBr): ν 3180 (NH), 1716 (CO), 1350 and 1550cm^−1 ^NO_2_. ^1^H-NMR (DMDO-d_6_): δ (ppm) 2.20 (s, 3H, CH_3_), 3.61 (s, 2H, CH_2_), 7.35 (m, 1H, H-4-phenyl), 7.73 (d, J=4 Hz, 2H, H-5,6-phenyl), 7.98 (d, J=8 Hz, 1H, H-3-phenyl), 10.50 (brs, 1H, NH).

*N-(2, 4-dinitrophenyl)-3-oxobutanamide (1f)*

Using the general procedure and 2,4-dinitroaniline provided the title compound after 4 h of reflux: Yellow crystals (from petroleum ether), Yield 68%, mp 162-165 °C. IR (KBr): ν 3300 (NH), 1726 (CO), 1350 and 1550cm^−1 ^NO_2_. ^1^H-NMR (DMDO-d_6_): δ (ppm) 2.17 (s, 3H, CH_3_), 3.51 (s, 2H, CH_2_), 8.01 (d, J-9.6Hz, 1H, H-6-phenyl), 8.52 (dd, J=9.6, 3.2 Hz, 1H, H-5-phenyl), 8.70 (d, J=3.2 Hz, 1H, H-3-phenyl), 10.71 (brs, 1H, NH).

*General procedure for preparation of diaryl 4-(4(5)-chloro-1H-imidazol-5 (4)-yl)-2, 6-dimethyl-1, 4-dihydropyridine-3, 5-di- carboxamide (3a-f)*

A light protected solution of compound 2 (1 mmol), ammonium acetate (1 mmol), and compounds 1a-f (2 mmol) in methanol (2.5 mL) was refluxed. The reaction mixture was cooled to room temperature, filtered and recrystallized from diethyl ether to give the title compounds.

*4-(4(5)-chloro-2-ethyl-1H-imidazol-5(4)-yl)-2, 6-dimethyl-N* ^3^ *, N* ^5^ *-bis (4-fluorophenyl)-1, 4-dihydropyridine-3, 5-dicarboxamide (3a)*

Using the general procedure and compound 1a provided the title compound after 24 h of reflux: white crystals, yield 56%; mp 240-247 ^o^C. IR (KBr): ν (cm^−1^) 3221 (NH), 1680, 1649(CO).; ^1^H-NMR (DMSO- *d*6): δ (ppm) 1.10 (t, J = 7.34 Hz, 3H, CH_3_CH_2_-), 2.09 (s, 6H, CH_3_-2,6-DHP), 2.48 (m, 2H, CH_3_CH_2_-), 5.12 (s, 1H, H-4-DHP), 7.10 (dd, J = 8.80, 8.96 Hz, 4H, H-3ʹ,5ʹ-phenyl), 7.49-7.67 (m, 4H, H-2ʹ,6ʹ-phenyl), 8.23 (s, 1H, HN-DHP), 9.23 (s, 2H, NH-amides), 11.36 (s, 1H, NH-imidazole).; Mass: m/z (rel.int.) 511(M^+^, 5), 509.3(6), 473.2(100), 404.3(22), 401.3(74), 365(45), 334.7(23), 271.1(47), 259.7(36), 158.7(21), 131.8(30), 129.8(37). Molecular Formula = C_26_H_24_ClF_2_N_5_O_2_; Calculated = C(61.00%) H(4.73%) N(13.68%), Found = C(61.05%) H(4.74%) N(13.70%).

*4-(4(5)-chloro-2-ethyl-1H-imidazol-5(4)-yl)-2, 6-dimethyl-N* ^3^ *, N* ^5^ *-bis (4-chlorophenyl)-1, 4-dihydropyridine-3, 5-dicarboxamide (3b)*

Using the general procedure and compound 1b provided the title compound after 24 h of reflux: White crystals, yield 58.5%; mp 249.5-252.2 ^o^C. IR (KBr): ν (cm^−1^) 3226 (NH), 1680, 1634 (CO). ^1^H-NMR (DMSO- *d*6): δ (ppm) 1.10 (t, J = 7.5Hz, 3H, CH_3_CH_2_-), 2.10 (s, 6H, CH_3_-2,6-DHP), 2.49 (m, 2H, CH_3_CH_2_-), 5.10 (s, 1H, H-4-DHP), 7.28 (d, J = 8.55 Hz, 4H, H-3ʹ,5ʹ-phenyl), 7.59 (d, J = 8.90Hz, 4H, H-2ʹ,6ʹ-phenyl), 8.39 (s, 1H, NH-DHP), 9.08 (s, 2H, NH-

amides), 11.32 (s, 1H, NH-imidazole).; Mass: m/z (rel.int.) 544(M^+^, 4), 510.1(30), 505.8(39), 436(23), 417(80), 380.7(100), 378.5(44), 286.9(29), 262.1(15), 154.9(8), 152.8(14), 127(22), 98.8(16). Molecular Formula = C_26_H_24_Cl_3_N_5_O_2_; Calculated = C(57.31%) H(4.44%) N(12.85%) , Found = C(57.39%) H(4.45%) N(12.83%).

*4-(4(5)-chloro-2-ethyl-1H-imidazol-5(4)-yl)-2, 6-dimethyl-N* ^3^ *, N5-bis (3,4-dichlorophenyl)-1, 4-dihydropyridine-3, 5-dicarboxamide (3c)*

Using the general procedure and compound 1c provided the title compound after 24 h of reflux: White crystals, yield 60.7%; mp 170.8-176.2 ^o^C. IR (KBr): ν (cm^−1^) 3298, 3262 (NH), 1654 (CO). ^1^H-NMR (DMSO- *d*6): δ (ppm) 1.08 (t, J = 7.33 Hz, 3H, CH_3_CH_2_-), 2.08 (s, 6H, CH_3_-2,6-DHP), 2.48 (m, 2H, CH_3_CH_2_-), 5.07 (s, 1H, H-4-DHP), 7.49 (s, 4H, H-5ʹ,6ʹ-phenyl), 7.97 (s, 2H, H-2ʹ-phenyl), 8.39 (s, 1H, NH-DHP), 9.48 (s, 2H, NH-amides), 11.32 (s, 1H, NH-imidazole).; Mass: m/z (rel.int.) 612.9(M^+^, 11), 575.8(3), 480(21), 414.8(8), 389(13), 320.7(87), 260.9(41), 186.8(63), 123.7(73), 106.2(100), 79.7(42). Molecular Formula = C_26_H_22_Cl_5_N_5_O_2_; Calculated = C(50.88%) H(3.61%)N(11.41%), Found = C(50.91%) H(3.62%)N(11.43%). 

*4-(4(5)-chloro-2-ethyl-1H-imidazol-5(4)-yl)-2, 6-dimethyl-N* ^3^ *, N5-bis (3-bromophenyl)-1, 4-dihydropyridine-3, 5-dicarboxamide (3d)*

Using the general procedure and compound 1d provided the title compound after 28 h of reflux: White crystals, yield 66%; mp 160.2-170.6 ^o^C. IR (KBr): ν (cm^−1^) 3308, 3277 (NH), 1640, 1634 (CO). ^1^H-NMR (DMSO- *d*6): δ (ppm) 1.12 (t, J=7.55 Hz, 3H, CH_3_CH_2_-), 2.12 (s, 6H, CH_3_-2,6-DHP), 2.52 (m, 2H, CH_3_CH_2_-), 5.12 (s, 1H, H-4-DHP), 7.2-7.3 (m, 4H, H-4ʹ,5ʹ-phenyl), 7.5-7.6 (m, 2H, H-6ʹ-phenyl), 7.8 (s, 2H, H-2ʹ-phenyl), 8.3 (s, 1H, NH-DHP), 9.4 (s, 2H, NH-amides), 11.3 (s, 1H, NH-imidazole).; Mass: m/z (rel.int.) 633(M^+^, 8), 598(15), 580(7), 504.6(23), 434.2(22), 333.1(31), 264.2(25), 235.9(37), 196.6(100), 132.1(78), 90.4(95). Molecular Formula = C_26_H_24_Br_2_ClN_5_O_2_; Calculated = C(49.27%) H(3.82%) N(11.05%), Found = C(49.24%) H(3.83%) N(11.03%).

*4-(4(5)-chloro-2-ethyl-1H-imidazol-5(4)-yl)-2, 6-dimethyl-N* ^3^ *, N5-bis (2-nitrophenyl)-1, 4-dihydropyridine-3, 5-dicarboxamide (3e)*

Using the general procedure and compound 1e provided the title compound after 32 h of reflux: Orange crystals, yield 68%; mp 180.1-190.2 ^o^C. IR (KBr): ν (cm^−1^) 3359, 3231 (NH), 1644 (CO), 1347, 1495(NO_2_). ^1^H-NMR (DMSO- *d*6): δ (ppm) 1.12 (t, J = 7.6 Hz, 3H, CH_3_CH_2_-), 2.18 (s, 6H, CH_3_-2,6-DHP), 2.51 (m, 2H, CH_3_CH_2_-), 5.01 (s, 1H, H-4-DHP), 7.28 (t, J = 7.72 Hz, 2H, H-4ʹ-phenyl), 7.68 (t, J = 7.72 Hz, 2H, H-5ʹ-phenyl) 7.78 (d, J = 7.65 Hz, 2H, H-6ʹ-phenyl), 7.98 (d, J = 8.25 Hz, 2H, H-3ʹ-phenyl), 8.63 (s, 1H, NH-DHP), 9.89 (s, 2H, NH-amides), 11.38 (s, 1H, NH-imidazole).; Mass: m/z (rel.int.) 565(M^+^, 7), 564.4(4), 528.1(10), 436.6 (22), 402(31), 400.4(85), 298.1(68), 232.7(100), 220.3(90), 164.4(65), 90(92), 63.3(92). Molecular Formula = C_26_H_24_ClN_7_O_6_; Calculated = C(55.18%) H(4.27%) N(17.32%), Found = C(55.23%) H(4.28%) N(17.34%).

*4-(4(5)-chloro-2-ethyl-1H-imidazol-5(4)-yl)-2, 6-dimethyl-N* ^3^ *, N5-bis (2,4-dinitrophenyl)-1, 4-dihydropyridine-3, 5-dicarboxamide (3f)*

Using the general procedure and compound 1f provided the title compound after 36 h of reflux: Yellow crystals, yield 62%; mp 139.5-143.3 ^o^C. IR (KBr): ν (cm^−1^) 3339(NH), 1639, 1680 (CO), 1337, 1265 (NO_2_). ^1^H-NMR (DMSO- *d*6): δ (ppm) 1.10 (t, J = 7.55 Hz, 3H, CH_3_CH_2_-), 2.27 (s, 6H, CH_3_-2,6-DHP), 2.57 (m, 2H, CH_3_CH_2_-), 5.06 (s, 1H, H-4-DHP), 8.06 (d, J=9.18 Hz, 2H, H-6ʹ-phenyl), 8.51 (dd, J = 9.06, 2.3 Hz, 2H, H-5ʹ-phenyl), 8.70(d, J = 2.32 Hz, 2H, H-3ʹ-phenyl), 8.97 (s, 1H, NH-DHP), 10.40 (s, 2H, NH-amides), 11.41 (s, 1H, NH-imidazole).; Mass: m/z (rel.int.) 469.9(6), 342.7(21), 287.9(48), 260.9(23), 245.9(16), 180.9(42), 107.2(29), 91.1(50), 62.1(75). 52.3 (100). Molecular Formula = C_26_H_22_ClN_9_O_10_; Calculated = C(47.61%) H(3.38%) N(19.22%), Found = C(47.66%) H(3.39%) N(19.25%).

*Computational studies*

The chemical structure of desired DHPs 3a-f was built and optimized using HYPERCHEM software (version 7, Hypercube Inc.). Optimization of the compounds was performed through MM+ and PM3 methods and total energy gradient was calculated as a root mean square (RMS) value, until the RMS gradient was 0.01 Kcal mol^-1^. The optimized conformer was transferred to Gaussian software to calculation of HOMO, LUMO and partial atomic charge (Muliken) using RHF method and 3-21G basis set.

*In-vitro evaluation of anti-mycobacterial activity*

The test compounds 3a-f, were initially dissolved in DMSO to give a concentration of 1 or 2 mg/L. All wells of micro plates received 100 µL of freshly prepared Middle broke 7H9 medium (Himedia, India), except first column. 200 µL of distilled water was added to the first column of 96 well plates to minimize evaporation of the medium in the test wells during incubation. Then 100 µL of test compounds with desired concentrations (1000 or 2000 µL) were added to the wells of the first row (each concentration was assayed in duplicate) and serial dilution was made from the first row to the last. Microbial suspension of BCG (1173P2) (100 µL), which had been prepared with standard concentration of 0.5 McFarland and diluted with 1:10 proportion by the distilled water, was added to all test wells. Plates were then sealed and incubated for 4 days at 37 °C. After that 12 µL Tween 80 10% and 20µL Alamar blue 0.01% (Himedia, India) were added to each test well. The results were assessed after 24 and 48 h. A blue color was interpreted as no bacterial growth, and color change to pink was scored as bacterial growth. Wells with a well-defined pink color were scored as positive for growth. The MIC (minimum inhibitory concentration) was defined as the lowest drug concentration, which prevented a color change from blue to pink. Ethambutol (Irandaru, Tehran) were used as positive control and DMSO as negative control (32).

## Results and Discussion

*Chemistry*

**Figure 2:**
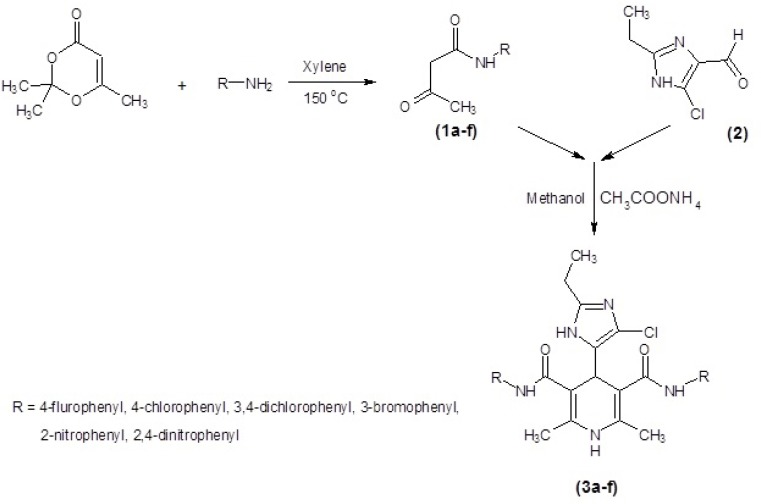
Synthesis of symmetrical DHPs 3a-f, by using classical Hantzsch condensation.

Six new derivatives of dihydropyridine, compounds 3a-f (Figure 2.), were synthesized using Hantzsch condensation in methanol at reflux condition and were purified by recrystallization in good yield (56% -68%). Structure of compounds characterized by TLC followed by IR, Mass, elemental analysis and proton NMR.

*Computational studies*

Based on the subjects that mentioned in the introduction section, Partial atomic charge (PAC) of carbon atom of carbonyl moiety at the C-3 and C-5 position of dihydropyridine ring and the lipophilicity (log p) of DHPs 3a-f was calculated using HyperChem and Gaussian software. To calculation the PAC, at first all of the compounds was optimized using HyperChem with molecular mechanics (MM+) and semi-empirical (PM3) methods. To finding the global minima, the best conformer from the previous stage was transferred to Gaussian and more optimization was performed using RHF method and 3-21G basis set. Confirmers with the global minima were used to calculation of partial atomic charges. Results of calculated PAC are presented in Table 2. Lipophilicity (log P) of designed compounds was calculated using HyeprChem (Table 2.). 

**Table 1:** Structure and *in-vitro* anti-tubercular activity of compounds 3a-f.

compound	R	Mp (°C)	Yield (%)	MIC(µm/ml) 24h	MIC(µm/ml) 48h
3a	4-flurophenyl	240-247	56	14.64	30.51
3b	4-chlorophenyl	249.5-252.2	58.5	<7.15	<7.15
3c	3,4-dichlorophenyl	170.8-176.2	60.7	<6.35	<6.35
3d	3-bromophenyl	160.2-170.6	66	11.83	24.64
3e	2-nitrophenyl	180.1-190.2	68	110.43	110.43
3f	2,4-dinitrophenyl	139.5-143.3	62	11.43	11.43
Ethambutol				3.04	3.04

**Table 2:** Calculated partial atomic charge (Muliken) of carbonyl groups at the C-3 and C-5 position of DHPs 3a-using Gaussian software

compound	C-3 carbonyl partial charge	C-5 carbonyl partial charge	Mean of charge of C-3 & C-5	Log p^a^
3a	0.765	0.793	0.779	1.43
3b	0.768	0.792	0.780	2.19
3c	0.785	0.801	0.793	3.22
3d	0.815	0.808	0.8115	2.74
3e	0.782	0.790	0.786	1.06
3f	0.845	0.811	0.828	0.97

Based on the results of the log p calculation (3c>3d>3b>3a>3c>3f), the compounds 3c and 3f are the more and less lipophilic ligands respectively. So it seems that the penetration of these ligands into the cell wall of mycobacterium is according to sequence of 3c>3d>3b>3a>3c>3f. PAC calculation shows in the designed compounds the carbon atom of carbonyl group at the C-3 and C-5 positions are efficiently positive, hence are susceptible to bio hydrolytic activation. Based on the results of mean of PAC at the C-3 and C-5 (3f>3d>3c>3e>3b>3a), the compounds 3f and 3a have the more and less positive PAC respectively. According to the PAC results compound 3f has high value of PAC, it is expected that compound 3f is more susceptible to enzymatic hydrolysis to produce the active compound and so it should be more potent than compound 3a. Considering both of parameters, compound 3f has the worse and the best value of logp and PAC respectively but fortunately the logp of this compound is still enough to penetration into the mycobacterium cells so it is expected to has a good biological activity. Compounds 3b, 3c and 3d have good values of both of parameters so it is expected they have very good potency against the TB.

*Anti-tubercular activity*

The ability of DHPs 3a-f to inhibition of mycobacterium tuberculosis growth was determined using *in-vitro* assay. The results are summarized in the Table 1. Each compound was dissolved in DMSO, Ethambutol and DMSO was used as positive and negative control respectively. The *in-vitro* screening data (Table1.) indicated that all analogs show a significant anti-tubercular activity in comparison to the reference drug ethambutol. Comparison of the MIC of compounds 3a (14.64, 30.51 µm/mL) and 3b (7.15 µm/mL) which contain F and Cl at the para position of phenyl ring with the same PAC (0.779 and 0.780 respectively), reveals that the compound 3b with more lipophilic characters’ is the more active than compounds 3a. Comparison of the MIC of compound 3e (110.4 µm/mL) with 3f (11.43 µm/mL) which contain one and two NO_2_ group respectively, show the importance of PAC in the anti-TB activity that the compound 3f with more amount of PAC is more potent than 3e. Finally comparison of MIC of compounds 3b (7.15 µm/mL) and 3c (6.35 µm/mL) which contain one and two Cl group respectively, show the importance of both of parameters (logp and PAC) on the anti-TB activity in which the compounds 3b and 3c are the most potent compounds. 

In comparison to DHPs which contain the N-substituted imidazole with an electron donating group (19), our pharmacological results have revealed that using the N-unsubstituted imidazole with an electron withdrawing group can efficiently increase the anti-tubercular activity. 

## Conclusion

Six DHPs analogs (3a-f) were synthesized and characterized by TLC followed by FT-IR, Mass and H^1^-NMR. The elemental analysis has confirmed the purity of products. Their ability to inhibition of mycobacterium growth was investigated in vitro evaluation. Based on the in vitro screening data all the designed and synthesized compounds 3a-f have the ability to inhibit the mycobacterium tuberculosis growth in term of MIC. The most potent compounds were 3b (4-Cl) and 3c (3,4-Cl), which our computational studies was predicted this in terms of PAC and logp.

The results so far indicated that the activity of these ligands against the mycobacterium can significantly be influenced by log p of molecules and partial atomic charge of carbon atom of carbonyl moiety at C-3 C-5 position of DHPs ring. The results have also confirmed that anti-tubercular activity can efficiently be increased using the N-unsubtituted imidazole with an electron withdrawing group. Currently, our research group is exploring this idea for designing newer ligands with better anti-tubercular activity. 
